# Aqua­dipyridine­bis­(*N*-*p*-tolysulfonyl-l-leucinato)nickel(II)

**DOI:** 10.1107/S1600536810038493

**Published:** 2010-10-02

**Authors:** Xi-Shi Tai

**Affiliations:** a Department of Chemistry, Weifangl University, Weifang 261061, People’s Republic of China

## Abstract

In each of the two mol­ecules in the asymmetric unit of the title compound, [Ni(C_13_H_18_NO_4_S)_2_(C_5_H_5_N)_2_(H_2_O)], the geometry of the Ni^2+^ ion is an extremely distorted *trans*-NiN_2_O_4_ octa­hedron, arising from its coordination by one water mol­ecule, two pyridine mol­ecules, and one *O*-monodentate and one *O*,*O*′-bidentate deprotonated *N*-*p*-tolysulfonyl-l-leucine ligand. In the crystal, mol­ecules are linked by N—H⋯O and O—H⋯O hydrogen bonds, forming chains along the *c* axis. An intra­molecular O—H⋯O hydrogen bond occurs in one of the mol­ecules.

## Related literature

For background to amino acid derivatives in coordination chemistry, see: Brown *et al.* (2008 ▶). For a related structure, see: Li *et al.* (2009 ▶).
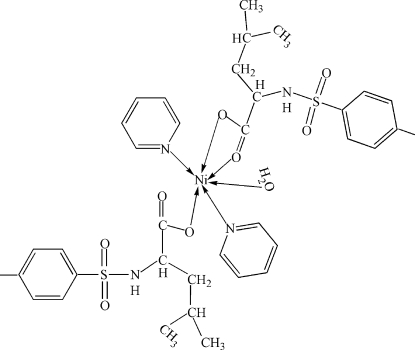


## Experimental

### 

#### Crystal data

[Ni(C_13_H_18_NO_4_S)_2_(C_5_H_5_N)_2_(H_2_O)]
*M* *_r_* = 803.61
Orthorhombic, 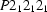

*a* = 18.1023 (16) Å
*b* = 19.5538 (19) Å
*c* = 22.956 (2) Å
*V* = 8125.7 (13) Å^3^
*Z* = 8
Mo *K*α radiation
μ = 0.64 mm^−1^
*T* = 298 K
0.38 × 0.36 × 0.31 mm

#### Data collection

Bruker SMART CCD diffractometer
Absorption correction: multi-scan (*SADABS*; Bruker, 2000 ▶) *T*_min_ = 0.794, *T*_max_ = 0.828
41421 measured reflections
14319 independent reflections
8009 reflections with *I* > 2σ(*I*)
*R*_int_ = 0.056

#### Refinement

*R*[*F* ^2^ > 2σ(*F* ^2^)] = 0.068
*wR*(*F* ^2^) = 0.242
*S* = 1.05
14319 reflections
949 parameters
H-atom parameters constrained
Δρ_max_ = 1.08 e Å^−3^
Δρ_min_ = −0.73 e Å^−3^
Absolute structure: Flack (1983 ▶), 6501 Friedel pairs
Flack parameter: −0.02 (3)

### 

Data collection: *SMART* (Bruker, 2000 ▶); cell refinement: *SAINT* (Bruker, 2000 ▶); data reduction: *SAINT*; program(s) used to solve structure: *SHELXS97* (Sheldrick, 2008 ▶); program(s) used to refine structure: *SHELXL97* (Sheldrick, 2008 ▶); molecular graphics: *SHELXTL* (Sheldrick, 2008 ▶); software used to prepare material for publication: *SHELXTL*.

## Supplementary Material

Crystal structure: contains datablocks global, I. DOI: 10.1107/S1600536810038493/hb5641sup1.cif

Structure factors: contains datablocks I. DOI: 10.1107/S1600536810038493/hb5641Isup2.hkl

Additional supplementary materials:  crystallographic information; 3D view; checkCIF report

## Figures and Tables

**Table 1:** Selected bond lengths (Å)

Ni1—O9	2.042 (5)
Ni1—O5	2.048 (6)
Ni1—N3	2.084 (7)
Ni1—N4	2.117 (7)
Ni1—O2	2.171 (6)
Ni1—O1	2.215 (6)
Ni2—O18	1.993 (5)
Ni2—O14	2.059 (6)
Ni2—N7	2.097 (7)
Ni2—N8	2.123 (7)
Ni2—O10	2.134 (6)
Ni2—O11	2.205 (5)

**Table 2:** Hydrogen-bond geometry (Å, °)

*D*—H⋯*A*	*D*—H	H⋯*A*	*D*⋯*A*	*D*—H⋯*A*
N1—H1⋯O15^i^	0.86	2.23	2.942 (10)	140
N2—H2⋯O10^i^	0.86	2.30	3.017 (9)	140
N5—H5⋯O2	0.86	2.43	3.144 (9)	141
O9—H9*C*⋯O14	0.85	2.31	2.754 (7)	113
O9—H9*D*⋯O11	0.85	1.87	2.610 (7)	145
O18—H18*E*⋯O6^ii^	0.85	1.74	2.569 (9)	165
O18—H18*F*⋯O15	0.85	2.00	2.683 (9)	136
O18—H18*F*⋯O1^ii^	0.85	2.37	2.820 (7)	113

Symmetry codes: (i) 
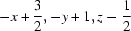
; (ii) 
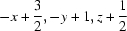
.

**Fig. 1.**
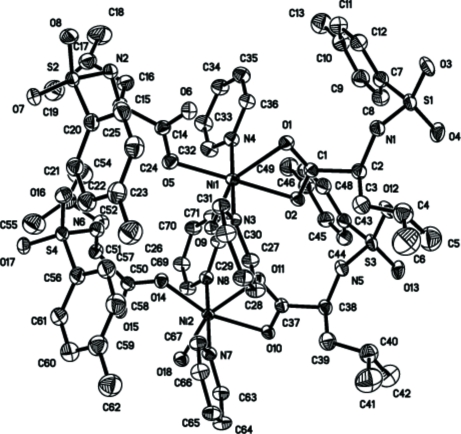
The molecular structure of (I) showing 30% displacement ellipsoids.

## supplementary crystallographic information

### Comment

Amino acid is an important for a class of physiological active substance, and the metal complexes of its small molecular ligands, not only are of important biological functions, but also are often essential active center that maintain structure and function of macromolecular organism complexes, such as metalloproteins and metal enzymes [Brown, *et al.*, 2008]. In this paper, we report on the synthesis and crystal structure of the title compound, (I), (Scheme I).

It can be seen that the coordination environment of the nickel atom consists of two nitrogen atoms from the two pyridine groups, three oxygen atoms from the two *N*-*p*-tolysulfonyl-L-leucine groups and one oxygen atom from the free water molecule, making up a distorted octahedral environment. The coordination oxygen atoms (O(1), O(2), O(5), O(9))and (O(10), O(11), O(14), O(18)) are situated equatorial place, and the nitrogen atoms are situated axial place. In addition, the four atoms (O(1), O(2), N(3), N(4)) are nearly coplanar. And the three atoms including (O(5), Ni(1), O(2)); (N(3), Ni(1), N(4)) and (N(7), Ni(2), N(8)) are nearly collinear. The distances of the Ni—O bonds are 1.993 (5)–2.215 (6)Å and Ni—N bonds are in the range of 2.084 (7)–2.123 (7)Å, respectively. They are similar to the Ni—O and Ni—N bond lengths reported previously [Li, *et al.*, 2009]. The dihedral angles between the benzene rings are 3.6 °, and between the pyridine rings are 3.7 °. In the crystal, the molecules form a one-dimensional chain structure by hydrogen bonds.

### Experimental

1 mmol (0.29 g) of nickel nitrate was added to the solution of pyridine (2 mmol, 0.16 g) in 15 ml of CH_3_OH/H_2_O (v:v=2:1). The mixture was continuously stirred for 1 h at refluxing temperature, then 2 mmol (0.56 g) of *N*-*p*-tolysulfonyl-L-leucine and 2 mmol(0.08 g) of sodium hydroxide were added to the mixture by stirring 6 h at refluxing temperature. The mixture was cooled at room temperature, and was collected by filtration. By evaporation in air at room temperature, light green blocks of (I) were obtained from methanol solution after two weeks. Yield 58%. Anal. Calcd. (%) for C_36_H_48_N_4_NiO_9_S_2_: C, 53.80; H, 6.07; N, 7.05. Found (%): C, 53.86; H, 6.10; N, 6.92.

### Crystal data

**Table:**

[Ni(C_13_H_18_NO_4_S)_2_(C_5_H_5_N)_2_(H_2_O)]	*D*_x_ = 1.314 Mg m^−^^3^
*M**_r_* = 803.61	Mo *K*α radiation, λ = 0.71073 Å
Orthorhombic, *P*2_1_2_1_2_1_	Cell parameters from 6010 reflections
*a* = 18.1023 (16) Å	θ = 2.4–23.6°
*b* = 19.5538 (19) Å	µ = 0.64 mm^−^^1^
*c* = 22.956 (2) Å	*T* = 298 K
*V* = 8125.7 (13) Å^3^	Block, light green
*Z* = 8	0.38 × 0.36 × 0.31 mm
*F*(000) = 3392	

### Data collection

**Table:**

Bruker SMART CCD diffractometer	14319 independent reflections
Radiation source: fine-focus sealed tube	8009 reflections with *I* > 2σ(*I*)
graphite	*R*_int_ = 0.056
phi and ω scans	θ_max_ = 25.0°, θ_min_ = 1.4°
Absorption correction: multi-scan (*SADABS*; Bruker, 2000)	*h* = −21→21
*T*_min_ = 0.794, *T*_max_ = 0.828	*k* = −12→23
41421 measured reflections	*l* = −27→26

### Refinement

**Table:**

Refinement on *F*^2^	Secondary atom site location: difference Fourier map
Least-squares matrix: full	Hydrogen site location: inferred from neighbouring sites
*R*[*F*^2^ > 2σ(*F*^2^)] = 0.068	H-atom parameters constrained
*wR*(*F*^2^) = 0.242	*w* = 1/[σ^2^(*F*_o_^2^) + (0.1133*P*)^2^ + 9.1777*P*] where *P* = (*F*_o_^2^ + 2*F*_c_^2^)/3
*S* = 1.05	(Δ/σ)_max_ = 0.001
14319 reflections	Δρ_max_ = 1.08 e Å^−^^3^
949 parameters	Δρ_min_ = −0.73 e Å^−^^3^
0 restraints	Absolute structure: Flack (1983), 6501 Friedel pairs
Primary atom site location: structure-invariant direct methods	Flack parameter: −0.02 (3)

### Special details

**Table:**

Geometry. All e.s.d.'s (except the e.s.d. in the dihedral angle between two l.s. planes) are estimated using the full covariance matrix. The cell e.s.d.'s are taken into account individually in the estimation of e.s.d.'s in distances, angles and torsion angles; correlations between e.s.d.'s in cell parameters are only used when they are defined by crystal symmetry. An approximate (isotropic) treatment of cell e.s.d.'s is used for estimating e.s.d.'s involving l.s. planes.
Refinement. Refinement of *F*^2^ against ALL reflections. The weighted *R*-factor *wR* and goodness of fit *S* are based on *F*^2^, conventional *R*-factors *R* are based on *F*, with *F* set to zero for negative *F*^2^. The threshold expression of *F*^2^ > σ(*F*^2^) is used only for calculating *R*-factors(gt) *etc*. and is not relevant to the choice of reflections for refinement. *R*-factors based on *F*^2^ are statistically about twice as large as those based on *F*, and *R*- factors based on ALL data will be even larger.

### Fractional atomic coordinates and isotropic or equivalent isotropic displacement parameters (Å^2^)

**Table:**

	*x*	*y*	*z*	*U*_iso_*/*U*_eq_	
Ni1	0.76523 (5)	0.50099 (6)	0.37915 (3)	0.0379 (3)	
Ni2	0.77376 (5)	0.49881 (6)	0.62033 (3)	0.0378 (3)	
N1	0.9600 (4)	0.4038 (4)	0.2580 (3)	0.055 (2)	
H1	0.9519	0.4330	0.2307	0.066*	
N2	0.5510 (4)	0.5974 (4)	0.2472 (3)	0.055 (2)	
H2	0.5540	0.5702	0.2178	0.066*	
N3	0.8263 (4)	0.5913 (3)	0.3807 (3)	0.0468 (17)	
N4	0.7028 (4)	0.4096 (4)	0.3743 (3)	0.0523 (19)	
N5	0.9634 (4)	0.3977 (3)	0.5003 (3)	0.0551 (17)	
H5	0.9559	0.4258	0.4721	0.066*	
N6	0.5690 (4)	0.5909 (4)	0.5067 (3)	0.066 (2)	
H6	0.5881	0.5642	0.4811	0.079*	
N7	0.8352 (4)	0.5896 (3)	0.6156 (3)	0.0476 (17)	
N8	0.7105 (4)	0.4077 (3)	0.6262 (3)	0.0504 (18)	
O1	0.8340 (3)	0.4698 (3)	0.3039 (2)	0.0519 (15)	
O2	0.8689 (3)	0.4478 (3)	0.3936 (2)	0.0482 (14)	
O3	0.9887 (4)	0.3277 (3)	0.1785 (3)	0.0734 (19)	
O4	1.0311 (3)	0.3015 (4)	0.2788 (3)	0.074 (2)	
O5	0.6665 (3)	0.5502 (3)	0.3671 (3)	0.0598 (16)	
O6	0.6880 (4)	0.5581 (4)	0.2726 (3)	0.0740 (19)	
O7	0.4767 (3)	0.6960 (3)	0.2772 (3)	0.0629 (17)	
O8	0.5203 (4)	0.6836 (3)	0.1747 (2)	0.0679 (18)	
O9	0.7479 (3)	0.5075 (3)	0.4669 (2)	0.0558 (15)	
H9C	0.7217	0.5418	0.4763	0.067*	
H9D	0.7873	0.5060	0.4870	0.067*	
O10	0.8760 (3)	0.4474 (3)	0.6354 (2)	0.0483 (14)	
O11	0.8378 (3)	0.4595 (3)	0.5458 (2)	0.0518 (15)	
O12	0.9871 (4)	0.3152 (3)	0.4238 (3)	0.0724 (19)	
O13	1.0341 (3)	0.2958 (3)	0.5240 (3)	0.0687 (19)	
O14	0.6879 (3)	0.5406 (3)	0.5735 (2)	0.0522 (14)	
O15	0.6206 (4)	0.5520 (4)	0.6533 (3)	0.0693 (19)	
O16	0.5394 (4)	0.6662 (4)	0.4244 (3)	0.075 (2)	
O17	0.4884 (3)	0.6901 (3)	0.5225 (3)	0.0680 (18)	
O18	0.7528 (3)	0.5277 (3)	0.7019 (2)	0.0598 (17)	
H18E	0.7645	0.4968	0.7263	0.072*	
H18F	0.7074	0.5375	0.7060	0.072*	
S1	0.97663 (14)	0.32680 (13)	0.24024 (9)	0.0567 (6)	
S2	0.53248 (13)	0.67640 (13)	0.23657 (9)	0.0528 (6)	
S3	0.97783 (13)	0.31938 (12)	0.48586 (9)	0.0574 (6)	
S4	0.54804 (13)	0.66715 (12)	0.48671 (9)	0.0593 (7)	
C1	0.8805 (5)	0.4480 (5)	0.3397 (4)	0.047 (2)	
C2	0.9577 (5)	0.4280 (5)	0.3194 (4)	0.057 (2)	
H2A	0.9793	0.3939	0.3456	0.068*	
C3	1.0035 (5)	0.5003 (6)	0.3233 (4)	0.076 (3)	
H3A	0.9790	0.5334	0.2983	0.092*	
H3B	1.0001	0.5172	0.3629	0.092*	
C4	1.0759 (6)	0.4979 (7)	0.3081 (5)	0.087 (3)	
H4	1.0796	0.4762	0.2698	0.105*	
C5	1.1199 (8)	0.4542 (8)	0.3521 (6)	0.124 (5)	
H5A	1.1324	0.4815	0.3854	0.187*	
H5B	1.1643	0.4379	0.3339	0.187*	
H5C	1.0904	0.4160	0.3642	0.187*	
C6	1.1148 (9)	0.5631 (9)	0.3057 (8)	0.154 (7)	
H6A	1.1031	0.5860	0.2698	0.231*	
H6B	1.1671	0.5552	0.3077	0.231*	
H6C	1.0998	0.5912	0.3379	0.231*	
C7	0.8968 (6)	0.2775 (6)	0.2514 (4)	0.066 (3)	
C8	0.8903 (6)	0.2361 (6)	0.2997 (5)	0.076 (3)	
H8	0.9273	0.2360	0.3279	0.092*	
C9	0.8279 (7)	0.1941 (6)	0.3065 (5)	0.084 (3)	
H9A	0.8234	0.1662	0.3392	0.101*	
C10	0.7732 (8)	0.1944 (6)	0.2645 (6)	0.083 (3)	
C11	0.7782 (7)	0.2371 (6)	0.2201 (5)	0.083 (3)	
H11	0.7394	0.2391	0.1937	0.099*	
C12	0.8407 (6)	0.2802 (6)	0.2110 (5)	0.075 (3)	
H12	0.8434	0.3091	0.1789	0.090*	
C13	0.7064 (8)	0.1472 (7)	0.2726 (6)	0.112 (5)	
H13A	0.6663	0.1630	0.2488	0.168*	
H13B	0.6915	0.1476	0.3128	0.168*	
H13C	0.7195	0.1015	0.2614	0.168*	
C14	0.6452 (5)	0.5585 (5)	0.3163 (4)	0.052 (2)	
C15	0.5630 (5)	0.5703 (5)	0.3062 (4)	0.053 (2)	
H15	0.5435	0.6022	0.3353	0.063*	
C16	0.5243 (5)	0.5001 (6)	0.3120 (4)	0.065 (2)	
H16A	0.5443	0.4697	0.2825	0.078*	
H16B	0.5369	0.4809	0.3497	0.078*	
C17	0.4420 (5)	0.5004 (6)	0.3064 (4)	0.073 (3)	
H17	0.4304	0.5248	0.2702	0.088*	
C18	0.4125 (8)	0.4287 (7)	0.2995 (6)	0.125 (5)	
H18A	0.4398	0.3982	0.3242	0.188*	
H18B	0.4175	0.4145	0.2597	0.188*	
H18C	0.3613	0.4278	0.3104	0.188*	
C19	0.4064 (7)	0.5381 (7)	0.3538 (6)	0.107 (4)	
H19A	0.4118	0.5132	0.3895	0.160*	
H19B	0.3549	0.5438	0.3452	0.160*	
H19C	0.4292	0.5822	0.3577	0.160*	
C20	0.6115 (6)	0.7264 (5)	0.2529 (4)	0.057 (3)	
C21	0.6153 (6)	0.7613 (5)	0.3054 (4)	0.065 (3)	
H21	0.5786	0.7555	0.3333	0.078*	
C22	0.6741 (6)	0.8049 (6)	0.3160 (5)	0.073 (3)	
H22	0.6761	0.8288	0.3510	0.088*	
C23	0.7299 (6)	0.8135 (6)	0.2754 (5)	0.074 (3)	
C24	0.7249 (7)	0.7769 (6)	0.2252 (5)	0.078 (3)	
H24	0.7623	0.7816	0.1977	0.094*	
C25	0.6673 (6)	0.7332 (5)	0.2131 (4)	0.068 (3)	
H25	0.6663	0.7087	0.1784	0.082*	
C26	0.7930 (7)	0.8635 (6)	0.2853 (6)	0.100 (4)	
H26A	0.7826	0.9056	0.2654	0.150*	
H26B	0.8380	0.8443	0.2705	0.150*	
H26C	0.7981	0.8722	0.3263	0.150*	
C27	0.8771 (5)	0.6018 (5)	0.4220 (4)	0.053 (2)	
H27	0.8819	0.5697	0.4516	0.064*	
C28	0.9234 (5)	0.6590 (5)	0.4227 (4)	0.061 (2)	
H28	0.9579	0.6647	0.4523	0.073*	
C29	0.9177 (5)	0.7059 (5)	0.3800 (4)	0.063 (3)	
H29	0.9472	0.7448	0.3798	0.076*	
C30	0.8667 (5)	0.6944 (5)	0.3366 (4)	0.063 (3)	
H30	0.8631	0.7250	0.3057	0.076*	
C31	0.8212 (5)	0.6382 (5)	0.3385 (4)	0.056 (2)	
H31	0.7857	0.6326	0.3096	0.067*	
C32	0.6552 (5)	0.3935 (5)	0.4169 (4)	0.066 (3)	
H32	0.6553	0.4197	0.4507	0.079*	
C33	0.6062 (6)	0.3402 (5)	0.4127 (5)	0.072 (3)	
H33	0.5741	0.3307	0.4433	0.086*	
C34	0.6050 (6)	0.3017 (5)	0.3642 (5)	0.068 (3)	
H34	0.5734	0.2644	0.3610	0.082*	
C35	0.6513 (6)	0.3188 (5)	0.3197 (5)	0.073 (3)	
H35	0.6505	0.2939	0.2852	0.087*	
C36	0.6993 (5)	0.3730 (5)	0.3258 (4)	0.057 (2)	
H36	0.7303	0.3842	0.2950	0.069*	
C37	0.8871 (5)	0.4423 (5)	0.5815 (4)	0.049 (2)	
C38	0.9624 (5)	0.4237 (5)	0.5604 (4)	0.057 (2)	
H38	0.9844	0.3898	0.5866	0.068*	
C39	1.0090 (5)	0.4940 (6)	0.5636 (4)	0.073 (3)	
H39A	0.9892	0.5255	0.5350	0.088*	
H39B	1.0017	0.5142	0.6018	0.088*	
C40	1.0858 (6)	0.4871 (6)	0.5538 (5)	0.085 (3)	
H40	1.0928	0.4610	0.5178	0.102*	
C41	1.1208 (8)	0.5564 (8)	0.5458 (7)	0.144 (6)	
H41A	1.1414	0.5596	0.5074	0.215*	
H41B	1.1592	0.5625	0.5741	0.215*	
H41C	1.0841	0.5913	0.5508	0.215*	
C42	1.1211 (8)	0.4503 (8)	0.6007 (6)	0.125 (5)	
H42A	1.1034	0.4672	0.6374	0.187*	
H42B	1.1736	0.4566	0.5984	0.187*	
H42C	1.1097	0.4025	0.5974	0.187*	
C43	0.8980 (5)	0.2718 (5)	0.5019 (5)	0.064 (2)	
C44	0.8944 (6)	0.2348 (5)	0.5536 (4)	0.072 (3)	
H44	0.9325	0.2374	0.5807	0.086*	
C45	0.8325 (6)	0.1936 (6)	0.5643 (5)	0.079 (3)	
H45	0.8300	0.1678	0.5982	0.095*	
C46	0.7745 (7)	0.1913 (6)	0.5239 (6)	0.082 (3)	
C47	0.7809 (7)	0.2284 (6)	0.4757 (5)	0.082 (3)	
H47	0.7428	0.2258	0.4486	0.098*	
C48	0.8397 (6)	0.2704 (6)	0.4628 (5)	0.076 (3)	
H48	0.8403	0.2969	0.4292	0.091*	
C49	0.7092 (7)	0.1442 (6)	0.5347 (6)	0.107 (4)	
H49A	0.6644	0.1670	0.5239	0.160*	
H49B	0.7073	0.1324	0.5753	0.160*	
H49C	0.7148	0.1034	0.5119	0.160*	
C50	0.6281 (5)	0.5530 (5)	0.5994 (4)	0.054 (2)	
C51	0.5579 (5)	0.5640 (5)	0.5645 (4)	0.062 (2)	
H51	0.5273	0.5968	0.5859	0.074*	
C52	0.5157 (6)	0.4939 (6)	0.5638 (5)	0.084 (3)	
H52A	0.5381	0.4652	0.5342	0.100*	
H52B	0.5237	0.4717	0.6010	0.100*	
C53	0.4375 (6)	0.4962 (7)	0.5532 (5)	0.095 (3)	
H53	0.4287	0.5197	0.5161	0.114*	
C54	0.4094 (9)	0.4213 (8)	0.5479 (8)	0.158 (7)	
H54A	0.4392	0.3920	0.5719	0.238*	
H54B	0.4128	0.4067	0.5081	0.238*	
H54C	0.3589	0.4189	0.5605	0.238*	
C55	0.3951 (8)	0.5313 (8)	0.5993 (7)	0.134 (6)	
H55A	0.4001	0.5066	0.6352	0.200*	
H55B	0.3439	0.5331	0.5884	0.200*	
H55C	0.4137	0.5769	0.6042	0.200*	
C56	0.6242 (5)	0.7204 (5)	0.5006 (5)	0.069 (3)	
C57	0.6807 (6)	0.7248 (6)	0.4614 (5)	0.080 (3)	
H57	0.6816	0.6975	0.4282	0.096*	
C58	0.7360 (7)	0.7708 (6)	0.4726 (5)	0.089 (4)	
H58	0.7739	0.7744	0.4454	0.106*	
C59	0.7395 (7)	0.8112 (6)	0.5203 (6)	0.087 (3)	
C60	0.6844 (7)	0.8042 (6)	0.5619 (5)	0.087 (3)	
H60	0.6866	0.8290	0.5965	0.105*	
C61	0.6254 (6)	0.7596 (6)	0.5515 (5)	0.077 (3)	
H61	0.5871	0.7559	0.5784	0.093*	
C62	0.8021 (7)	0.8594 (6)	0.5310 (6)	0.111 (4)	
H62A	0.8435	0.8466	0.5073	0.166*	
H62B	0.8160	0.8576	0.5714	0.166*	
H62C	0.7870	0.9051	0.5213	0.166*	
C63	0.8813 (5)	0.6052 (5)	0.6601 (4)	0.055 (2)	
H63	0.8847	0.5751	0.6913	0.065*	
C64	0.9227 (5)	0.6632 (5)	0.6610 (4)	0.062 (3)	
H64	0.9538	0.6721	0.6924	0.074*	
C65	0.9184 (5)	0.7078 (5)	0.6160 (4)	0.062 (3)	
H65	0.9459	0.7479	0.6164	0.074*	
C66	0.8738 (5)	0.6932 (5)	0.5706 (4)	0.066 (3)	
H66	0.8719	0.7220	0.5384	0.079*	
C67	0.8309 (5)	0.6345 (5)	0.5727 (4)	0.056 (2)	
H67	0.7976	0.6264	0.5426	0.067*	
C68	0.6698 (5)	0.3942 (5)	0.6721 (4)	0.061 (2)	
H68	0.6750	0.4218	0.7049	0.073*	
C69	0.6190 (6)	0.3405 (5)	0.6742 (4)	0.065 (3)	
H69	0.5928	0.3314	0.7082	0.078*	
C70	0.6083 (5)	0.3013 (5)	0.6253 (4)	0.065 (3)	
H70	0.5738	0.2661	0.6243	0.078*	
C71	0.6514 (6)	0.3169 (5)	0.5778 (5)	0.068 (3)	
H71	0.6469	0.2913	0.5438	0.082*	
C72	0.7004 (5)	0.3692 (5)	0.5799 (4)	0.057 (2)	
H72	0.7284	0.3783	0.5469	0.068*	

### Atomic displacement parameters (Å^2^)

**Table:**

	*U*^11^	*U*^22^	*U*^33^	*U*^12^	*U*^13^	*U*^23^
Ni1	0.0456 (5)	0.0384 (6)	0.0299 (5)	−0.0012 (6)	−0.0009 (4)	0.0023 (5)
Ni2	0.0463 (5)	0.0375 (5)	0.0296 (5)	0.0033 (7)	−0.0035 (4)	0.0000 (5)
N1	0.060 (5)	0.063 (5)	0.044 (4)	0.014 (4)	0.001 (3)	0.002 (4)
N2	0.060 (5)	0.057 (5)	0.048 (4)	0.013 (4)	−0.005 (3)	−0.005 (4)
N3	0.053 (4)	0.044 (4)	0.044 (4)	−0.002 (3)	−0.004 (4)	−0.002 (3)
N4	0.055 (4)	0.049 (5)	0.053 (5)	−0.006 (4)	0.004 (4)	0.000 (4)
N5	0.065 (4)	0.057 (4)	0.043 (4)	0.019 (3)	0.006 (4)	0.004 (4)
N6	0.070 (5)	0.068 (5)	0.061 (5)	0.018 (4)	−0.011 (4)	−0.008 (5)
N7	0.056 (4)	0.045 (4)	0.042 (4)	0.001 (3)	−0.002 (4)	0.001 (3)
N8	0.057 (4)	0.049 (5)	0.046 (4)	0.001 (4)	−0.002 (4)	0.004 (3)
O1	0.054 (3)	0.052 (4)	0.049 (3)	0.003 (3)	−0.005 (3)	0.000 (3)
O2	0.055 (3)	0.050 (4)	0.039 (3)	0.005 (3)	0.005 (3)	−0.001 (3)
O3	0.086 (5)	0.074 (5)	0.060 (4)	0.031 (4)	0.019 (4)	−0.010 (4)
O4	0.064 (4)	0.086 (5)	0.074 (4)	0.030 (4)	−0.002 (4)	0.014 (4)
O5	0.061 (4)	0.055 (4)	0.063 (4)	0.002 (3)	−0.012 (3)	0.002 (3)
O6	0.064 (4)	0.085 (5)	0.074 (5)	0.019 (4)	−0.005 (4)	−0.012 (4)
O7	0.055 (4)	0.070 (4)	0.064 (4)	0.017 (3)	0.003 (3)	−0.015 (3)
O8	0.092 (5)	0.067 (4)	0.044 (3)	0.019 (4)	−0.018 (3)	0.001 (3)
O9	0.052 (3)	0.072 (4)	0.043 (3)	0.013 (3)	−0.007 (2)	−0.009 (3)
O10	0.056 (3)	0.052 (4)	0.036 (3)	0.006 (3)	0.008 (3)	0.001 (3)
O11	0.054 (3)	0.059 (4)	0.043 (3)	0.009 (3)	0.002 (3)	0.001 (3)
O12	0.098 (5)	0.075 (5)	0.044 (3)	0.029 (4)	0.014 (3)	−0.006 (3)
O13	0.060 (4)	0.074 (4)	0.072 (4)	0.028 (3)	0.002 (3)	0.016 (3)
O14	0.051 (3)	0.057 (4)	0.049 (3)	0.006 (3)	0.000 (3)	0.005 (3)
O15	0.071 (4)	0.086 (5)	0.051 (4)	0.028 (4)	0.004 (3)	0.004 (4)
O16	0.087 (5)	0.091 (5)	0.047 (4)	0.027 (4)	−0.017 (3)	0.001 (4)
O17	0.061 (4)	0.080 (5)	0.063 (4)	0.027 (4)	−0.002 (3)	−0.010 (3)
O18	0.063 (4)	0.068 (4)	0.048 (3)	0.001 (3)	0.008 (3)	−0.003 (3)
S1	0.0578 (15)	0.0663 (17)	0.0459 (13)	0.0231 (13)	0.0074 (11)	0.0025 (11)
S2	0.0584 (14)	0.0570 (15)	0.0429 (13)	0.0214 (12)	−0.0021 (10)	−0.0054 (10)
S3	0.0683 (14)	0.0610 (15)	0.0431 (13)	0.0325 (12)	0.0091 (11)	0.0051 (11)
S4	0.0666 (14)	0.0648 (15)	0.0465 (14)	0.0265 (12)	−0.0127 (11)	−0.0076 (11)
C1	0.052 (5)	0.046 (5)	0.043 (5)	0.002 (4)	0.003 (4)	0.000 (4)
C2	0.056 (5)	0.069 (6)	0.046 (5)	0.008 (5)	0.001 (4)	−0.001 (5)
C3	0.072 (6)	0.086 (7)	0.070 (6)	0.003 (7)	0.006 (5)	−0.008 (7)
C4	0.086 (8)	0.091 (8)	0.084 (7)	−0.003 (8)	0.009 (6)	−0.002 (8)
C5	0.116 (11)	0.137 (13)	0.120 (12)	0.008 (10)	0.007 (10)	0.001 (11)
C6	0.134 (14)	0.149 (16)	0.179 (17)	−0.041 (13)	0.013 (13)	−0.001 (14)
C7	0.070 (7)	0.070 (7)	0.058 (6)	0.017 (6)	−0.004 (5)	−0.003 (6)
C8	0.078 (8)	0.083 (8)	0.068 (7)	0.005 (7)	−0.002 (6)	0.002 (6)
C9	0.088 (8)	0.085 (8)	0.079 (8)	0.002 (7)	−0.001 (7)	0.000 (6)
C10	0.083 (8)	0.079 (9)	0.088 (9)	0.006 (7)	−0.001 (8)	−0.006 (7)
C11	0.081 (8)	0.086 (9)	0.080 (8)	0.003 (7)	−0.006 (7)	−0.008 (7)
C12	0.078 (7)	0.080 (8)	0.068 (7)	0.011 (6)	−0.005 (6)	−0.002 (6)
C13	0.109 (11)	0.099 (10)	0.129 (12)	−0.010 (9)	−0.005 (9)	−0.003 (9)
C14	0.057 (6)	0.052 (6)	0.048 (5)	0.001 (5)	−0.007 (5)	0.001 (5)
C15	0.049 (5)	0.058 (6)	0.052 (5)	0.006 (4)	−0.009 (4)	−0.002 (4)
C16	0.064 (5)	0.064 (6)	0.066 (5)	−0.003 (6)	−0.007 (4)	−0.001 (6)
C17	0.067 (6)	0.081 (7)	0.072 (6)	−0.005 (7)	−0.006 (5)	−0.005 (7)
C18	0.111 (11)	0.131 (13)	0.134 (12)	−0.045 (10)	−0.016 (9)	−0.021 (10)
C19	0.083 (8)	0.123 (11)	0.114 (10)	−0.014 (8)	0.002 (8)	−0.008 (9)
C20	0.060 (7)	0.057 (6)	0.055 (6)	0.014 (5)	0.001 (4)	0.002 (5)
C21	0.067 (6)	0.068 (7)	0.060 (6)	0.009 (6)	0.001 (5)	0.002 (5)
C22	0.076 (7)	0.076 (7)	0.068 (7)	0.010 (6)	−0.001 (6)	0.001 (6)
C23	0.071 (7)	0.070 (7)	0.081 (7)	0.004 (6)	0.003 (7)	0.012 (6)
C24	0.076 (8)	0.083 (8)	0.076 (7)	0.007 (7)	0.013 (7)	0.011 (7)
C25	0.071 (7)	0.072 (7)	0.062 (6)	0.012 (6)	0.007 (5)	0.001 (5)
C26	0.092 (9)	0.095 (9)	0.113 (10)	−0.012 (8)	−0.003 (8)	0.004 (8)
C27	0.059 (5)	0.055 (6)	0.046 (5)	−0.006 (5)	−0.005 (5)	0.001 (5)
C28	0.068 (6)	0.060 (6)	0.055 (6)	−0.015 (5)	−0.007 (5)	−0.003 (5)
C29	0.069 (6)	0.060 (7)	0.061 (6)	−0.018 (5)	0.000 (5)	−0.001 (5)
C30	0.070 (6)	0.060 (7)	0.059 (6)	−0.013 (5)	−0.002 (5)	0.010 (5)
C31	0.061 (6)	0.055 (6)	0.051 (5)	−0.005 (5)	−0.006 (4)	0.003 (5)
C32	0.075 (7)	0.062 (7)	0.060 (6)	−0.014 (5)	0.011 (5)	−0.003 (5)
C33	0.079 (7)	0.068 (7)	0.069 (7)	−0.021 (6)	0.012 (6)	0.003 (6)
C34	0.070 (7)	0.060 (7)	0.075 (7)	−0.022 (5)	0.002 (6)	−0.001 (6)
C35	0.079 (7)	0.066 (7)	0.074 (7)	−0.007 (6)	0.002 (6)	−0.006 (6)
C36	0.063 (6)	0.055 (6)	0.055 (6)	−0.006 (5)	0.003 (5)	0.001 (5)
C37	0.055 (5)	0.051 (5)	0.042 (5)	0.006 (4)	0.004 (4)	0.004 (4)
C38	0.060 (6)	0.063 (6)	0.047 (5)	0.013 (5)	0.003 (4)	0.003 (4)
C39	0.073 (6)	0.075 (7)	0.071 (6)	0.003 (7)	0.008 (5)	0.002 (7)
C40	0.080 (7)	0.090 (10)	0.084 (8)	−0.004 (7)	0.007 (6)	0.003 (7)
C41	0.130 (12)	0.145 (14)	0.156 (14)	−0.050 (12)	0.007 (12)	0.014 (12)
C42	0.111 (11)	0.140 (13)	0.122 (12)	−0.011 (10)	−0.010 (10)	0.013 (11)
C43	0.075 (6)	0.062 (6)	0.056 (5)	0.021 (5)	−0.010 (6)	−0.006 (6)
C44	0.080 (7)	0.069 (7)	0.066 (7)	0.010 (6)	−0.006 (6)	0.000 (6)
C45	0.085 (8)	0.076 (8)	0.076 (7)	0.006 (6)	−0.010 (6)	−0.005 (6)
C46	0.079 (8)	0.075 (8)	0.092 (9)	0.007 (7)	−0.010 (7)	−0.009 (7)
C47	0.087 (9)	0.081 (8)	0.077 (8)	0.006 (7)	−0.015 (7)	−0.014 (7)
C48	0.082 (8)	0.076 (8)	0.070 (7)	0.016 (7)	−0.008 (6)	−0.004 (6)
C49	0.095 (9)	0.098 (10)	0.128 (11)	−0.011 (8)	−0.004 (8)	−0.008 (8)
C50	0.060 (6)	0.053 (6)	0.049 (6)	0.006 (5)	−0.002 (5)	−0.004 (4)
C51	0.057 (5)	0.060 (6)	0.067 (6)	0.008 (5)	−0.013 (5)	−0.002 (5)
C52	0.076 (7)	0.076 (8)	0.098 (8)	0.003 (7)	−0.014 (6)	−0.011 (7)
C53	0.090 (8)	0.092 (9)	0.103 (9)	−0.006 (9)	−0.007 (7)	−0.013 (9)
C54	0.154 (15)	0.141 (15)	0.180 (17)	−0.048 (13)	0.003 (13)	−0.032 (13)
C55	0.119 (12)	0.145 (15)	0.137 (13)	−0.031 (10)	0.013 (10)	−0.020 (11)
C56	0.069 (6)	0.075 (7)	0.062 (6)	0.019 (5)	−0.007 (6)	−0.001 (6)
C57	0.081 (8)	0.088 (8)	0.072 (7)	0.013 (7)	−0.002 (6)	−0.003 (6)
C58	0.087 (9)	0.096 (10)	0.083 (8)	0.007 (8)	0.002 (7)	0.006 (7)
C59	0.085 (8)	0.083 (9)	0.093 (9)	0.005 (7)	−0.009 (7)	0.005 (7)
C60	0.086 (8)	0.086 (9)	0.089 (9)	0.010 (7)	−0.006 (7)	−0.008 (7)
C61	0.077 (7)	0.082 (8)	0.072 (7)	0.012 (6)	−0.003 (6)	−0.009 (6)
C62	0.103 (10)	0.101 (10)	0.128 (12)	−0.012 (8)	−0.007 (9)	−0.002 (8)
C63	0.064 (6)	0.051 (6)	0.049 (5)	−0.006 (5)	−0.005 (5)	0.005 (4)
C64	0.067 (6)	0.061 (7)	0.057 (6)	−0.019 (5)	−0.012 (5)	−0.001 (5)
C65	0.068 (6)	0.057 (6)	0.060 (6)	−0.016 (5)	−0.003 (5)	−0.003 (5)
C66	0.072 (6)	0.063 (7)	0.063 (6)	−0.008 (5)	−0.004 (5)	0.008 (5)
C67	0.063 (6)	0.056 (6)	0.048 (5)	−0.006 (5)	−0.006 (5)	0.004 (5)
C68	0.071 (6)	0.059 (6)	0.052 (6)	−0.011 (5)	0.000 (5)	0.000 (5)
C69	0.077 (7)	0.060 (7)	0.059 (6)	−0.016 (5)	0.003 (5)	0.008 (5)
C70	0.073 (7)	0.059 (6)	0.064 (7)	−0.018 (5)	−0.003 (5)	0.000 (5)
C71	0.078 (7)	0.061 (7)	0.065 (6)	−0.013 (6)	−0.005 (6)	−0.007 (5)
C72	0.065 (6)	0.056 (6)	0.049 (5)	−0.006 (5)	0.000 (5)	−0.006 (5)

### Geometric parameters (Å, °)

**Table:**

Ni1—O9	2.042 (5)	C22—C23	1.386 (15)
Ni1—O5	2.048 (6)	C22—H22	0.9300
Ni1—N3	2.084 (7)	C23—C24	1.359 (15)
Ni1—N4	2.117 (7)	C23—C26	1.520 (15)
Ni1—O2	2.171 (6)	C24—C25	1.376 (15)
Ni1—O1	2.215 (6)	C24—H24	0.9300
Ni2—O18	1.993 (5)	C25—H25	0.9300
Ni2—O14	2.059 (6)	C26—H26A	0.9600
Ni2—N7	2.097 (7)	C26—H26B	0.9600
Ni2—N8	2.123 (7)	C26—H26C	0.9600
Ni2—O10	2.134 (6)	C27—C28	1.398 (12)
Ni2—O11	2.205 (5)	C27—H27	0.9300
N1—C2	1.488 (10)	C28—C29	1.345 (12)
N1—S1	1.588 (8)	C28—H28	0.9300
N1—H1	0.8600	C29—C30	1.376 (12)
N2—C15	1.471 (10)	C29—H29	0.9300
N2—S2	1.600 (7)	C30—C31	1.376 (12)
N2—H2	0.8600	C30—H30	0.9300
N3—C31	1.336 (10)	C31—H31	0.9300
N3—C27	1.338 (10)	C32—C33	1.372 (13)
N4—C36	1.325 (10)	C32—H32	0.9300
N4—C32	1.341 (11)	C33—C34	1.345 (13)
N5—C38	1.471 (11)	C33—H33	0.9300
N5—S3	1.589 (7)	C34—C35	1.363 (13)
N5—H5	0.8600	C34—H34	0.9300
N6—C51	1.441 (12)	C35—C36	1.377 (13)
N6—S4	1.605 (7)	C35—H35	0.9300
N6—H6	0.8600	C36—H36	0.9300
N7—C67	1.323 (10)	C37—C38	1.492 (12)
N7—C63	1.354 (10)	C38—C39	1.613 (13)
N8—C68	1.313 (10)	C38—H38	0.9800
N8—C72	1.316 (10)	C39—C40	1.415 (14)
O1—C1	1.251 (10)	C39—H39A	0.9700
O2—C1	1.254 (9)	C39—H39B	0.9700
O3—S1	1.434 (6)	C40—C42	1.443 (16)
O4—S1	1.413 (6)	C40—C41	1.508 (17)
O5—C14	1.239 (10)	C40—H40	0.9800
O6—C14	1.268 (10)	C41—H41A	0.9600
O7—S2	1.427 (6)	C41—H41B	0.9600
O8—S2	1.444 (6)	C41—H41C	0.9600
O9—H9C	0.8501	C42—H42A	0.9600
O9—H9D	0.8500	C42—H42B	0.9600
O10—C37	1.259 (9)	C42—H42C	0.9600
O11—C37	1.256 (10)	C43—C48	1.384 (13)
O12—S3	1.436 (6)	C43—C44	1.392 (13)
O13—S3	1.420 (6)	C44—C45	1.401 (14)
O14—C50	1.259 (10)	C44—H44	0.9300
O15—C50	1.245 (10)	C45—C46	1.401 (15)
O16—S4	1.439 (6)	C45—H45	0.9300
O17—S4	1.429 (6)	C46—C47	1.327 (14)
O18—H18E	0.8500	C46—C49	1.519 (16)
O18—H18F	0.8500	C47—C48	1.377 (15)
S1—C7	1.757 (12)	C47—H47	0.9300
S2—C20	1.773 (11)	C48—H48	0.9300
S3—C43	1.758 (10)	C49—H49A	0.9600
S4—C56	1.758 (11)	C49—H49B	0.9600
C1—C2	1.525 (12)	C49—H49C	0.9600
C2—C3	1.640 (14)	C50—C51	1.518 (12)
C2—H2A	0.9800	C51—C52	1.570 (14)
C3—C4	1.357 (14)	C51—H51	0.9800
C3—H3A	0.9700	C52—C53	1.438 (14)
C3—H3B	0.9700	C52—H52A	0.9700
C4—C6	1.458 (18)	C52—H52B	0.9700
C4—C5	1.544 (17)	C53—C55	1.476 (16)
C4—H4	0.9800	C53—C54	1.555 (18)
C5—H5A	0.9600	C53—H53	0.9800
C5—H5B	0.9600	C54—H54A	0.9600
C5—H5C	0.9600	C54—H54B	0.9600
C6—H6A	0.9600	C54—H54C	0.9600
C6—H6B	0.9600	C55—H55A	0.9600
C6—H6C	0.9600	C55—H55B	0.9600
C7—C12	1.377 (14)	C55—H55C	0.9600
C7—C8	1.377 (14)	C56—C57	1.365 (14)
C8—C9	1.406 (15)	C56—C61	1.397 (14)
C8—H8	0.9300	C57—C58	1.370 (15)
C9—C10	1.383 (16)	C57—H57	0.9300
C9—H9A	0.9300	C58—C59	1.353 (15)
C10—C11	1.321 (15)	C58—H58	0.9300
C10—C13	1.531 (17)	C59—C60	1.388 (16)
C11—C12	1.426 (15)	C59—C62	1.495 (16)
C11—H11	0.9300	C60—C61	1.400 (15)
C12—H12	0.9300	C60—H60	0.9300
C13—H13A	0.9600	C61—H61	0.9300
C13—H13B	0.9600	C62—H62A	0.9600
C13—H13C	0.9600	C62—H62B	0.9600
C14—C15	1.523 (12)	C62—H62C	0.9600
C15—C16	1.547 (13)	C63—C64	1.359 (12)
C15—H15	0.9800	C63—H63	0.9300
C16—C17	1.494 (12)	C64—C65	1.355 (12)
C16—H16A	0.9700	C64—H64	0.9300
C16—H16B	0.9700	C65—C66	1.349 (12)
C17—C19	1.465 (15)	C65—H65	0.9300
C17—C18	1.508 (16)	C66—C67	1.387 (12)
C17—H17	0.9800	C66—H66	0.9300
C18—H18A	0.9600	C67—H67	0.9300
C18—H18B	0.9600	C68—C69	1.397 (13)
C18—H18C	0.9600	C68—H68	0.9300
C19—H19A	0.9600	C69—C70	1.373 (13)
C19—H19B	0.9600	C69—H69	0.9300
C19—H19C	0.9600	C70—C71	1.375 (13)
C20—C25	1.369 (13)	C70—H70	0.9300
C20—C21	1.386 (13)	C71—C72	1.353 (12)
C21—C22	1.386 (14)	C71—H71	0.9300
C21—H21	0.9300	C72—H72	0.9300
			
O9—Ni1—O5	88.3 (2)	C24—C23—C22	117.3 (11)
O9—Ni1—N3	90.7 (3)	C24—C23—C26	121.1 (11)
O5—Ni1—N3	93.8 (3)	C22—C23—C26	121.6 (11)
O9—Ni1—N4	91.3 (3)	C23—C24—C25	123.2 (11)
O5—Ni1—N4	85.6 (3)	C23—C24—H24	118.4
N3—Ni1—N4	177.9 (3)	C25—C24—H24	118.4
O9—Ni1—O2	90.7 (2)	C20—C25—C24	119.0 (10)
O5—Ni1—O2	178.8 (2)	C20—C25—H25	120.5
N3—Ni1—O2	86.8 (3)	C24—C25—H25	120.5
N4—Ni1—O2	93.8 (3)	C23—C26—H26A	109.5
O9—Ni1—O1	150.8 (2)	C23—C26—H26B	109.5
O5—Ni1—O1	120.9 (2)	H26A—C26—H26B	109.5
N3—Ni1—O1	87.1 (2)	C23—C26—H26C	109.5
N4—Ni1—O1	91.5 (2)	H26A—C26—H26C	109.5
O2—Ni1—O1	60.1 (2)	H26B—C26—H26C	109.5
O18—Ni2—O14	103.6 (2)	N3—C27—C28	122.9 (9)
O18—Ni2—N7	84.8 (2)	N3—C27—H27	118.6
O14—Ni2—N7	92.1 (2)	C28—C27—H27	118.6
O18—Ni2—N8	94.3 (3)	C29—C28—C27	119.4 (9)
O14—Ni2—N8	87.7 (2)	C29—C28—H28	120.3
N7—Ni2—N8	179.1 (3)	C27—C28—H28	120.3
O18—Ni2—O10	98.4 (2)	C28—C29—C30	117.9 (9)
O14—Ni2—O10	157.8 (2)	C28—C29—H29	121.1
N7—Ni2—O10	87.0 (2)	C30—C29—H29	121.1
N8—Ni2—O10	93.5 (2)	C31—C30—C29	120.6 (9)
O18—Ni2—O11	158.1 (2)	C31—C30—H30	119.7
O14—Ni2—O11	97.5 (2)	C29—C30—H30	119.7
N7—Ni2—O11	88.6 (2)	N3—C31—C30	122.0 (8)
N8—Ni2—O11	92.3 (2)	N3—C31—H31	119.0
O10—Ni2—O11	60.4 (2)	C30—C31—H31	119.0
C2—N1—S1	123.4 (6)	N4—C32—C33	122.9 (10)
C2—N1—H1	118.3	N4—C32—H32	118.6
S1—N1—H1	118.3	C33—C32—H32	118.6
C15—N2—S2	121.3 (6)	C34—C33—C32	119.6 (10)
C15—N2—H2	119.4	C34—C33—H33	120.2
S2—N2—H2	119.4	C32—C33—H33	120.2
C31—N3—C27	117.1 (7)	C33—C34—C35	118.2 (9)
C31—N3—Ni1	122.1 (6)	C33—C34—H34	120.9
C27—N3—Ni1	120.5 (6)	C35—C34—H34	120.9
C36—N4—C32	117.0 (8)	C34—C35—C36	120.1 (10)
C36—N4—Ni1	121.8 (6)	C34—C35—H35	120.0
C32—N4—Ni1	120.2 (6)	C36—C35—H35	120.0
C38—N5—S3	122.1 (6)	N4—C36—C35	122.1 (9)
C38—N5—H5	118.9	N4—C36—H36	118.9
S3—N5—H5	118.9	C35—C36—H36	118.9
C51—N6—S4	124.7 (7)	O11—C37—O10	120.4 (8)
C51—N6—H6	117.6	O11—C37—C38	120.2 (8)
S4—N6—H6	117.6	O10—C37—C38	118.9 (8)
C67—N7—C63	116.6 (8)	N5—C38—C37	113.5 (7)
C67—N7—Ni2	124.7 (6)	N5—C38—C39	109.3 (7)
C63—N7—Ni2	118.6 (6)	C37—C38—C39	104.9 (7)
C68—N8—C72	117.1 (8)	N5—C38—H38	109.7
C68—N8—Ni2	121.5 (6)	C37—C38—H38	109.7
C72—N8—Ni2	120.3 (6)	C39—C38—H38	109.7
C1—O1—Ni1	87.7 (5)	C40—C39—C38	115.2 (10)
C1—O2—Ni1	89.6 (5)	C40—C39—H39A	108.5
C14—O5—Ni1	117.5 (6)	C38—C39—H39A	108.5
Ni1—O9—H9C	112.7	C40—C39—H39B	108.5
Ni1—O9—H9D	113.8	C38—C39—H39B	108.5
H9C—O9—H9D	110.9	H39A—C39—H39B	107.5
C37—O10—Ni2	90.9 (5)	C39—C40—C42	111.3 (11)
C37—O11—Ni2	87.8 (5)	C39—C40—C41	110.3 (11)
C50—O14—Ni2	118.5 (5)	C42—C40—C41	110.7 (12)
Ni2—O18—H18E	111.7	C39—C40—H40	108.1
Ni2—O18—H18F	110.6	C42—C40—H40	108.1
H18E—O18—H18F	109.2	C41—C40—H40	108.1
O4—S1—O3	121.1 (4)	C40—C41—H41A	109.5
O4—S1—N1	107.6 (4)	C40—C41—H41B	109.5
O3—S1—N1	105.7 (4)	H41A—C41—H41B	109.5
O4—S1—C7	106.9 (5)	C40—C41—H41C	109.5
O3—S1—C7	106.0 (5)	H41A—C41—H41C	109.5
N1—S1—C7	109.1 (5)	H41B—C41—H41C	109.5
O7—S2—O8	120.5 (4)	C40—C42—H42A	109.5
O7—S2—N2	107.9 (4)	C40—C42—H42B	109.5
O8—S2—N2	106.0 (4)	H42A—C42—H42B	109.5
O7—S2—C20	106.6 (4)	C40—C42—H42C	109.5
O8—S2—C20	106.1 (4)	H42A—C42—H42C	109.5
N2—S2—C20	109.4 (4)	H42B—C42—H42C	109.5
O13—S3—O12	120.6 (4)	C48—C43—C44	120.4 (10)
O13—S3—N5	107.6 (4)	C48—C43—S3	120.1 (9)
O12—S3—N5	106.3 (4)	C44—C43—S3	119.5 (8)
O13—S3—C43	106.8 (4)	C43—C44—C45	119.1 (10)
O12—S3—C43	105.9 (5)	C43—C44—H44	120.5
N5—S3—C43	109.3 (4)	C45—C44—H44	120.5
O17—S4—O16	119.6 (4)	C44—C45—C46	120.1 (11)
O17—S4—N6	107.8 (4)	C44—C45—H45	119.9
O16—S4—N6	107.4 (4)	C46—C45—H45	119.9
O17—S4—C56	107.6 (5)	C47—C46—C45	117.9 (12)
O16—S4—C56	105.8 (5)	C47—C46—C49	122.4 (12)
N6—S4—C56	108.3 (4)	C45—C46—C49	119.6 (12)
O1—C1—O2	122.5 (8)	C46—C47—C48	124.9 (12)
O1—C1—C2	120.2 (7)	C46—C47—H47	117.5
O2—C1—C2	117.0 (8)	C48—C47—H47	117.5
N1—C2—C1	113.3 (7)	C47—C48—C43	117.5 (11)
N1—C2—C3	108.2 (7)	C47—C48—H48	121.3
C1—C2—C3	103.1 (7)	C43—C48—H48	121.3
N1—C2—H2A	110.7	C46—C49—H49A	109.5
C1—C2—H2A	110.7	C46—C49—H49B	109.5
C3—C2—H2A	110.7	H49A—C49—H49B	109.5
C4—C3—C2	116.4 (11)	C46—C49—H49C	109.5
C4—C3—H3A	108.2	H49A—C49—H49C	109.5
C2—C3—H3A	108.2	H49B—C49—H49C	109.5
C4—C3—H3B	108.2	O15—C50—O14	124.1 (8)
C2—C3—H3B	108.2	O15—C50—C51	115.8 (9)
H3A—C3—H3B	107.3	O14—C50—C51	119.8 (8)
C3—C4—C6	116.5 (13)	N6—C51—C50	115.0 (8)
C3—C4—C5	110.5 (11)	N6—C51—C52	112.1 (8)
C6—C4—C5	105.1 (12)	C50—C51—C52	106.8 (8)
C3—C4—H4	108.2	N6—C51—H51	107.6
C6—C4—H4	108.2	C50—C51—H51	107.6
C5—C4—H4	108.2	C52—C51—H51	107.6
C4—C5—H5A	109.5	C53—C52—C51	117.0 (10)
C4—C5—H5B	109.5	C53—C52—H52A	108.0
H5A—C5—H5B	109.5	C51—C52—H52A	108.0
C4—C5—H5C	109.5	C53—C52—H52B	108.0
H5A—C5—H5C	109.5	C51—C52—H52B	108.0
H5B—C5—H5C	109.5	H52A—C52—H52B	107.3
C4—C6—H6A	109.5	C52—C53—C55	113.9 (11)
C4—C6—H6B	109.5	C52—C53—C54	107.8 (12)
H6A—C6—H6B	109.5	C55—C53—C54	108.9 (12)
C4—C6—H6C	109.5	C52—C53—H53	108.7
H6A—C6—H6C	109.5	C55—C53—H53	108.7
H6B—C6—H6C	109.5	C54—C53—H53	108.7
C12—C7—C8	120.2 (11)	C53—C54—H54A	109.5
C12—C7—S1	119.1 (9)	C53—C54—H54B	109.5
C8—C7—S1	120.7 (9)	H54A—C54—H54B	109.5
C7—C8—C9	120.0 (11)	C53—C54—H54C	109.5
C7—C8—H8	120.0	H54A—C54—H54C	109.5
C9—C8—H8	120.0	H54B—C54—H54C	109.5
C10—C9—C8	119.8 (12)	C53—C55—H55A	109.5
C10—C9—H9A	120.1	C53—C55—H55B	109.5
C8—C9—H9A	120.1	H55A—C55—H55B	109.5
C11—C10—C9	119.4 (13)	C53—C55—H55C	109.5
C11—C10—C13	121.9 (13)	H55A—C55—H55C	109.5
C9—C10—C13	118.6 (12)	H55B—C55—H55C	109.5
C10—C11—C12	122.9 (12)	C57—C56—C61	120.5 (11)
C10—C11—H11	118.6	C57—C56—S4	120.3 (9)
C12—C11—H11	118.6	C61—C56—S4	119.2 (9)
C7—C12—C11	117.5 (11)	C56—C57—C58	117.7 (11)
C7—C12—H12	121.2	C56—C57—H57	121.1
C11—C12—H12	121.2	C58—C57—H57	121.1
C10—C13—H13A	109.5	C59—C58—C57	124.6 (12)
C10—C13—H13B	109.5	C59—C58—H58	117.7
H13A—C13—H13B	109.5	C57—C58—H58	117.7
C10—C13—H13C	109.5	C58—C59—C60	117.9 (12)
H13A—C13—H13C	109.5	C58—C59—C62	122.5 (13)
H13B—C13—H13C	109.5	C60—C59—C62	119.5 (12)
O5—C14—O6	123.5 (9)	C59—C60—C61	119.5 (12)
O5—C14—C15	117.9 (9)	C59—C60—H60	120.3
O6—C14—C15	118.6 (8)	C61—C60—H60	120.3
N2—C15—C14	109.8 (7)	C56—C61—C60	119.7 (11)
N2—C15—C16	109.4 (7)	C56—C61—H61	120.2
C14—C15—C16	107.2 (7)	C60—C61—H61	120.2
N2—C15—H15	110.1	C59—C62—H62A	109.5
C14—C15—H15	110.1	C59—C62—H62B	109.5
C16—C15—H15	110.1	H62A—C62—H62B	109.5
C17—C16—C15	116.2 (9)	C59—C62—H62C	109.5
C17—C16—H16A	108.2	H62A—C62—H62C	109.5
C15—C16—H16A	108.2	H62B—C62—H62C	109.5
C17—C16—H16B	108.2	N7—C63—C64	122.7 (8)
C15—C16—H16B	108.2	N7—C63—H63	118.6
H16A—C16—H16B	107.4	C64—C63—H63	118.6
C19—C17—C16	112.1 (9)	C65—C64—C63	119.5 (9)
C19—C17—C18	112.9 (11)	C65—C64—H64	120.2
C16—C17—C18	111.1 (11)	C63—C64—H64	120.2
C19—C17—H17	106.8	C66—C65—C64	119.2 (9)
C16—C17—H17	106.8	C66—C65—H65	120.4
C18—C17—H17	106.8	C64—C65—H65	120.4
C17—C18—H18A	109.5	C65—C66—C67	118.9 (9)
C17—C18—H18B	109.5	C65—C66—H66	120.6
H18A—C18—H18B	109.5	C67—C66—H66	120.6
C17—C18—H18C	109.5	N7—C67—C66	122.8 (9)
H18A—C18—H18C	109.5	N7—C67—H67	118.6
H18B—C18—H18C	109.5	C66—C67—H67	118.6
C17—C19—H19A	109.5	N8—C68—C69	123.2 (9)
C17—C19—H19B	109.5	N8—C68—H68	118.4
H19A—C19—H19B	109.5	C69—C68—H68	118.4
C17—C19—H19C	109.5	C70—C69—C68	119.0 (9)
H19A—C19—H19C	109.5	C70—C69—H69	120.5
H19B—C19—H19C	109.5	C68—C69—H69	120.5
C25—C20—C21	119.7 (10)	C69—C70—C71	116.4 (9)
C25—C20—S2	120.5 (8)	C69—C70—H70	121.8
C21—C20—S2	119.7 (8)	C71—C70—H70	121.8
C22—C21—C20	119.6 (10)	C72—C71—C70	120.7 (9)
C22—C21—H21	120.2	C72—C71—H71	119.6
C20—C21—H21	120.2	C70—C71—H71	119.6
C23—C22—C21	121.1 (11)	N8—C72—C71	123.5 (9)
C23—C22—H22	119.5	N8—C72—H72	118.2
C21—C22—H22	119.5	C71—C72—H72	118.2
			
O9—Ni1—N3—C31	−140.7 (7)	S2—N2—C15—C16	138.4 (7)
O5—Ni1—N3—C31	−52.3 (7)	O5—C14—C15—N2	164.5 (8)
N4—Ni1—N3—C31	22 (8)	O6—C14—C15—N2	−15.0 (12)
O2—Ni1—N3—C31	128.7 (7)	O5—C14—C15—C16	−76.7 (10)
O1—Ni1—N3—C31	68.5 (7)	O6—C14—C15—C16	103.8 (10)
O9—Ni1—N3—C27	45.0 (6)	N2—C15—C16—C17	−63.7 (10)
O5—Ni1—N3—C27	133.3 (6)	C14—C15—C16—C17	177.3 (8)
N4—Ni1—N3—C27	−152 (7)	C15—C16—C17—C19	−65.2 (12)
O2—Ni1—N3—C27	−45.7 (6)	C15—C16—C17—C18	167.5 (9)
O1—Ni1—N3—C27	−105.8 (6)	O7—S2—C20—C25	−162.6 (8)
O9—Ni1—N4—C36	−172.3 (7)	O8—S2—C20—C25	−33.0 (9)
O5—Ni1—N4—C36	99.5 (7)	N2—S2—C20—C25	81.0 (9)
N3—Ni1—N4—C36	25 (8)	O7—S2—C20—C21	14.6 (9)
O2—Ni1—N4—C36	−81.6 (7)	O8—S2—C20—C21	144.2 (8)
O1—Ni1—N4—C36	−21.4 (7)	N2—S2—C20—C21	−101.9 (8)
O9—Ni1—N4—C32	19.5 (7)	C25—C20—C21—C22	2.6 (15)
O5—Ni1—N4—C32	−68.7 (7)	S2—C20—C21—C22	−174.6 (8)
N3—Ni1—N4—C32	−143 (7)	C20—C21—C22—C23	−1.0 (16)
O2—Ni1—N4—C32	110.3 (7)	C21—C22—C23—C24	−0.7 (16)
O1—Ni1—N4—C32	170.4 (7)	C21—C22—C23—C26	177.3 (10)
O18—Ni2—N7—C67	−132.4 (7)	C22—C23—C24—C25	0.9 (17)
O14—Ni2—N7—C67	−28.9 (7)	C26—C23—C24—C25	−177.1 (10)
N8—Ni2—N7—C67	−108 (17)	C21—C20—C25—C24	−2.5 (15)
O10—Ni2—N7—C67	128.9 (7)	S2—C20—C25—C24	174.7 (8)
O11—Ni2—N7—C67	68.5 (7)	C23—C24—C25—C20	0.7 (17)
O18—Ni2—N7—C63	45.5 (6)	C31—N3—C27—C28	0.5 (13)
O14—Ni2—N7—C63	149.0 (6)	Ni1—N3—C27—C28	175.1 (7)
N8—Ni2—N7—C63	70 (17)	N3—C27—C28—C29	−0.4 (14)
O10—Ni2—N7—C63	−53.2 (6)	C27—C28—C29—C30	−1.2 (14)
O11—Ni2—N7—C63	−113.6 (6)	C28—C29—C30—C31	2.8 (15)
O18—Ni2—N8—C68	7.0 (7)	C27—N3—C31—C30	1.2 (13)
O14—Ni2—N8—C68	−96.5 (7)	Ni1—N3—C31—C30	−173.4 (7)
N7—Ni2—N8—C68	−18 (17)	C29—C30—C31—N3	−2.8 (15)
O10—Ni2—N8—C68	105.7 (7)	C36—N4—C32—C33	2.4 (14)
O11—Ni2—N8—C68	166.1 (7)	Ni1—N4—C32—C33	171.1 (8)
O18—Ni2—N8—C72	174.6 (7)	N4—C32—C33—C34	−0.1 (16)
O14—Ni2—N8—C72	71.1 (7)	C32—C33—C34—C35	−2.1 (16)
N7—Ni2—N8—C72	150 (17)	C33—C34—C35—C36	2.0 (16)
O10—Ni2—N8—C72	−86.7 (7)	C32—N4—C36—C35	−2.6 (14)
O11—Ni2—N8—C72	−26.3 (7)	Ni1—N4—C36—C35	−171.1 (7)
O9—Ni1—O1—C1	−0.8 (8)	C34—C35—C36—N4	0.4 (15)
O5—Ni1—O1—C1	178.0 (5)	Ni2—O11—C37—O10	6.8 (8)
N3—Ni1—O1—C1	85.3 (5)	Ni2—O11—C37—C38	−165.5 (8)
N4—Ni1—O1—C1	−96.2 (5)	Ni2—O10—C37—O11	−7.1 (9)
O2—Ni1—O1—C1	−2.7 (5)	Ni2—O10—C37—C38	165.4 (7)
O9—Ni1—O2—C1	−176.4 (5)	S3—N5—C38—C37	−108.0 (8)
O5—Ni1—O2—C1	151 (11)	S3—N5—C38—C39	135.3 (6)
N3—Ni1—O2—C1	−85.7 (5)	O11—C37—C38—N5	−26.3 (12)
N4—Ni1—O2—C1	92.3 (5)	O10—C37—C38—N5	161.2 (8)
O1—Ni1—O2—C1	2.7 (5)	O11—C37—C38—C39	92.9 (10)
O9—Ni1—O5—C14	−176.8 (7)	O10—C37—C38—C39	−79.5 (10)
N3—Ni1—O5—C14	92.6 (7)	N5—C38—C39—C40	−67.0 (11)
N4—Ni1—O5—C14	−85.4 (7)	C37—C38—C39—C40	171.1 (9)
O2—Ni1—O5—C14	−144 (11)	C38—C39—C40—C42	−68.1 (13)
O1—Ni1—O5—C14	3.7 (7)	C38—C39—C40—C41	168.6 (10)
O18—Ni2—O10—C37	−170.5 (5)	O13—S3—C43—C48	−163.5 (8)
O14—Ni2—O10—C37	2.1 (9)	O12—S3—C43—C48	−33.8 (9)
N7—Ni2—O10—C37	−86.2 (5)	N5—S3—C43—C48	80.4 (9)
N8—Ni2—O10—C37	94.6 (5)	O13—S3—C43—C44	15.4 (9)
O11—Ni2—O10—C37	4.0 (5)	O12—S3—C43—C44	145.1 (8)
O18—Ni2—O11—C37	10.9 (9)	N5—S3—C43—C44	−100.7 (8)
O14—Ni2—O11—C37	175.3 (5)	C48—C43—C44—C45	3.0 (15)
N7—Ni2—O11—C37	83.3 (5)	S3—C43—C44—C45	−176.0 (8)
N8—Ni2—O11—C37	−96.8 (5)	C43—C44—C45—C46	−1.5 (16)
O10—Ni2—O11—C37	−4.0 (5)	C44—C45—C46—C47	0.8 (17)
O18—Ni2—O14—C50	−24.8 (7)	C44—C45—C46—C49	177.5 (10)
N7—Ni2—O14—C50	−110.0 (7)	C45—C46—C47—C48	−1.5 (18)
N8—Ni2—O14—C50	69.1 (7)	C49—C46—C47—C48	−178.1 (11)
O10—Ni2—O14—C50	162.8 (7)	C46—C47—C48—C43	3.0 (17)
O11—Ni2—O14—C50	161.1 (6)	C44—C43—C48—C47	−3.6 (15)
C2—N1—S1—O4	−37.8 (8)	S3—C43—C48—C47	175.3 (8)
C2—N1—S1—O3	−168.5 (7)	Ni2—O14—C50—O15	12.0 (13)
C2—N1—S1—C7	77.8 (8)	Ni2—O14—C50—C51	−162.4 (6)
C15—N2—S2—O7	−46.4 (8)	S4—N6—C51—C50	−108.8 (9)
C15—N2—S2—O8	−176.8 (7)	S4—N6—C51—C52	129.0 (8)
C15—N2—S2—C20	69.1 (8)	O15—C50—C51—N6	157.2 (8)
C38—N5—S3—O13	−40.1 (8)	O14—C50—C51—N6	−28.0 (13)
C38—N5—S3—O12	−170.6 (7)	O15—C50—C51—C52	−77.8 (11)
C38—N5—S3—C43	75.6 (8)	O14—C50—C51—C52	97.0 (10)
C51—N6—S4—O17	−27.6 (8)	N6—C51—C52—C53	−75.9 (12)
C51—N6—S4—O16	−157.7 (7)	C50—C51—C52—C53	157.4 (10)
C51—N6—S4—C56	88.5 (8)	C51—C52—C53—C55	−65.0 (16)
Ni1—O1—C1—O2	4.8 (9)	C51—C52—C53—C54	174.1 (11)
Ni1—O1—C1—C2	−168.0 (8)	O17—S4—C56—C57	−159.9 (8)
Ni1—O2—C1—O1	−4.9 (9)	O16—S4—C56—C57	−31.0 (9)
Ni1—O2—C1—C2	168.1 (7)	N6—S4—C56—C57	83.8 (9)
S1—N1—C2—C1	−111.1 (8)	O17—S4—C56—C61	18.5 (9)
S1—N1—C2—C3	135.2 (7)	O16—S4—C56—C61	147.4 (8)
O1—C1—C2—N1	−28.9 (12)	N6—S4—C56—C61	−97.8 (8)
O2—C1—C2—N1	157.9 (8)	C61—C56—C57—C58	−2.9 (16)
O1—C1—C2—C3	87.8 (9)	S4—C56—C57—C58	175.5 (8)
O2—C1—C2—C3	−85.4 (9)	C56—C57—C58—C59	1.4 (18)
N1—C2—C3—C4	−60.5 (12)	C57—C58—C59—C60	2.1 (19)
C1—C2—C3—C4	179.2 (9)	C57—C58—C59—C62	178.6 (11)
C2—C3—C4—C6	173.9 (11)	C58—C59—C60—C61	−4.1 (18)
C2—C3—C4—C5	−66.3 (13)	C62—C59—C60—C61	179.3 (11)
O4—S1—C7—C12	−165.3 (8)	C57—C56—C61—C60	0.9 (16)
O3—S1—C7—C12	−34.8 (10)	S4—C56—C61—C60	−177.5 (8)
N1—S1—C7—C12	78.6 (9)	C59—C60—C61—C56	2.7 (17)
O4—S1—C7—C8	14.3 (10)	C67—N7—C63—C64	−1.3 (13)
O3—S1—C7—C8	144.7 (9)	Ni2—N7—C63—C64	−179.4 (7)
N1—S1—C7—C8	−101.8 (9)	N7—C63—C64—C65	0.0 (15)
C12—C7—C8—C9	3.0 (17)	C63—C64—C65—C66	−1.0 (15)
S1—C7—C8—C9	−176.5 (8)	C64—C65—C66—C67	3.1 (15)
C7—C8—C9—C10	0.1 (17)	C63—N7—C67—C66	3.6 (13)
C8—C9—C10—C11	−3.9 (18)	Ni2—N7—C67—C66	−178.4 (7)
C8—C9—C10—C13	179.1 (10)	C65—C66—C67—N7	−4.6 (15)
C9—C10—C11—C12	4.6 (18)	C72—N8—C68—C69	2.0 (14)
C13—C10—C11—C12	−178.4 (11)	Ni2—N8—C68—C69	170.0 (7)
C8—C7—C12—C11	−2.4 (16)	N8—C68—C69—C70	−2.9 (15)
S1—C7—C12—C11	177.2 (8)	C68—C69—C70—C71	2.3 (15)
C10—C11—C12—C7	−1.5 (17)	C69—C70—C71—C72	−1.1 (15)
Ni1—O5—C14—O6	−22.3 (12)	C68—N8—C72—C71	−0.7 (14)
Ni1—O5—C14—C15	158.2 (6)	Ni2—N8—C72—C71	−168.8 (7)
S2—N2—C15—C14	−104.3 (8)	C70—C71—C72—N8	0.3 (15)

### Hydrogen-bond geometry (Å, °)

**Table:**

*D*—H···*A*	*D*—H	H···*A*	*D*···*A*	*D*—H···*A*
N1—H1···O15^i^	0.86	2.23	2.942 (10)	140
N2—H2···O10^i^	0.86	2.30	3.017 (9)	140
N5—H5···O2	0.86	2.43	3.144 (9)	141
O9—H9C···O14	0.85	2.31	2.754 (7)	113
O9—H9D···O11	0.85	1.87	2.610 (7)	145
O18—H18E···O6^ii^	0.85	1.74	2.569 (9)	165
O18—H18F···O15	0.85	2.00	2.683 (9)	136
O18—H18F···O1^ii^	0.85	2.37	2.820 (7)	113

Symmetry codes: (i) −*x*+3/2, −*y*+1, *z*−1/2; (ii) −*x*+3/2, −*y*+1, *z*+1/2.
